# Investigation into the Role of PI3K and JAK3 Kinase Inhibitors in Murine Models of Asthma

**DOI:** 10.3389/fphar.2017.00082

**Published:** 2017-02-28

**Authors:** Akshaya D. Wagh, Manoranjan Sharma, Jogeshwar Mahapatra, Abhijeet Chatterjee, Mukul Jain, Veeranjaneyulu Addepalli

**Affiliations:** ^1^Department of Pharmacology, SPP SPTM, SVKM's NMIMSMumbai, India; ^2^Department of Pharmacology, Zydus Research CentreAhmedabad, India

**Keywords:** asthma, eosinophil infiltration, cytokine, ovalbumin, kinase inhibitor

## Abstract

Asthma is a clinical disorder commonly characterized by chronic eosinophilic inflammation, remodeling and hyper responsiveness of the airways. However, the kinases like Phosphoinositide 3 kinase (PI3K) and Janus kinase 3 (JAK3) are involved in mast cell proliferation, activation, recruitment, migration, and prolonged survival of inflammatory cells. The present study was designed to evaluate the *in-vivo* comparative effects of two kinase inhibitors on airway inflammation and airway remodeling in acute and chronic models of asthma. Mice were sensitized twice intra-peritoneally and then challenged by inhalation with ovalbumin (OVA). They developed an extensive inflammatory response, goblet cell hyperplasia, collagen deposition, airway smooth muscle thickening similar to pathologies observed in human asthma. The effects of PI3K inhibitor (30 mg/kg, p.o), JAK3 inhibitor (30 mg/kg, p.o) and Dexamethasone (0.3 mg/kg) on airway inflammation and remodeling in OVA sensitized/challenged BALB/c mice were evaluated. Twenty-four hours after the final antigen challenge, bronchoalveolar lavage (BAL) and histological examinations were carried out. It was observed that kinase inhibitors significantly reduced airway inflammation as evidenced by the decrease in pro inflammatory cytokines in BALF and lung homogenate and inflammatory cell count in sensitized mice after allergen challenge. Lung histological analysis showed increased infiltration of inflammatory cells, hyperplasia of goblet cells and the collagen deposition, which were significantly reduced with kinase inhibitor. In conclusion, our data suggest that PI3K and JAK3 inhibitors showed promising alternative therapeutic activity in asthma, which might significantly counteract the airway inflammation in patients with allergic asthma.

## Introduction

Asthma is a complex disease characterized clinically by reversible bronchial obstruction (Deshpande et al., 2010) and airway hyper-responsiveness (AHR) (Brannan and Lougheed, 2012). Asthma affects more than 334 million people worldwide (GAR, 2014). Post-mortem and lung biopsy studies have revealed pulmonary infiltration of eosinophils, lymphocytes, macrophages, and mast cells in asthmatics. In addition, structural changes in the cellular and extracellular components of the asthmatic bronchi, such as airway wall thickening, subepithelial fibrosis, goblet cell hyperplasia, myofibroblast hyperplasia, airway smooth muscle cell hyperplasia, hypertrophy, and epithelial hypertrophy collectively which are known as airway remodeling have been reported (Szefler and Dakhama, 2011). Allergic asthma is a common inflammatory disease of the airway, and long-term therapy is aimed to counteract the episodes of bronchospasm and reducing allergic inflammation (Montuschi and Barnes, 2011). Although such strategies are successful, they neither cure nor prevent asthma and in some cases, have not prevented the disease from progressing (Colice et al., 2012). Therefore, new therapeutic strategies must be identified to overcome from limited drug availability (Holgate et al., 2008). Thus, there is an unmet need to develop new targeted therapy with improved efficacy and safety profile. Generally, targeting a single cell may not be an ideal approach for the treatment of asthma because of the redundant and overlapping responses of inflammatory cells. Therefore, a more plausible approach must be considered to target the common upstream pathway that controls the pathogenic responses of multiple inflammatory components.

Kinase signaling cascades represent an attractive target for anti-inflammatory therapy for asthma. Specific inhibitors reduce activity of critical kinase signaling cascade, which might produce sufficient relief to patients suffering from allergic airway inflammation. Among these Janus kinase 3 (JAK3) has been reported to regulate macrophage expression by inducible nitric oxide synthase (iNOS) and nitric oxide production (Mullen and Gonzalez-Perez, 2016), mast cell proliferation and functioning (Han et al., 2014). JAK3 is also involved in immunoglobin-E (IgE) class switching, mediating responses of dendritic cells, macrophages and non-hematopoietic lineage cells in asthma (Malaviya and Laskin, 2010; Walford and Doherty, 2013). JAK3 inhibitors act as potent inhibitors of IgE receptor-mediated 5-lipooxygenase (LO) dependent leukotriene synthesis in mast cells. JAK3 inhibitor indirectly inhibits the enzymatic activity of 5-LO, by preventing the antigen/IgE dependent translocation of 5-LO from nucleoplasm/cytoplasm to nuclear envelope (Liao et al., 2013). Phosphoinositide 3 kinase (PI3K) inhibitors revealed their anti-inflammatory effects by suppressing antigen-induced airway inflammatory cell infiltration, production of interleukin-4 (IL-4), interleukin-5 (IL-5), interleukin-13 (IL-13), eosinophil cationic protein, and eotaxin in BAL fluid, goblet cell hyperplasia, and airway hyper responsiveness (Stark et al., 2015). Hence this study has been carried out to investigate the comparitive role of PI3 Kinase and Janus Associated Kinase 3 inhibitors as novel therapies for the treatment of asthma.

## Materials and methods

### Experimental animal

Seven- to eight- weeks- old BALB/c mice were purchased and maintained in the specific pathogen-free animal facility at Zydus Research Centre (Gujarat, India). For the acute study, the mice (*n* = 6 per group) were then sensitized intraperitoneally on day 0 with 2%OVA (Qualigens fine chemicals) and 1% alum in normal saline (0.2 ml per mice) and 5% OVA and 1% alum on day 7. The mice were periodically challenged with 5% OVA for 30 min through nebulizer from days 14 to 16 in an acrylic chamber. On day 17 (24 h after the final OVA challenge) the mice were sacrificed. In the chronic study, mice were intraperitoneally injected with 2% OVA and 1% of Alum on day 0, followed by 5% OVA and 1% alum on day 14. The same mice were challenged with 5% OVA from days 21 to 30 (Donaldson et al., 2013). On day 31, the mice were sacrificed and BAL and lungs were collected. As a negative control, saline was used instead of OVA during the sensitization and challenge phase, for both acute and chronic study. All animal experimental protocols were approved by the Zydus Animal Ethics Committee.

### Treatment

The mice were treated with PI3K inhibitor (INK654 molecule obtained from Intellikine Inc, 30 mg/kg), JAK3 inhibitor (Tofacitinib, Pifzer, 30 mg/kg) and dexamethasone (0.3 mg/kg). The drugs were prepared in 0.5% carboxymethylcellulose and administered orally 1 h prior to OVA challenge–from days 14 to 16 (3 days) and days 21 to 30 (10 days) for acute and chronic study, respectively. Control mice received vehicle orally. All drugs were freshly prepared. The inflammatory cell counts and cytokines levels were measured 24 h after the final OVA challenge. Cytokines were measured in BAL and lung homogenate by using ELISA reagent kit.

### Bronchoalveolar lavage

Bronchoalveolar lavage (BAL) was immediately performed after 24 h of final OVA challenge. Mice were sacrificed by spinal dislocation. The lungs were lavaged *insitu* via tracheal cannula with ice-cold heparinised saline (0.5 ml X 4) followed by centrifugation of BALF (8000 rpm for 10 min at 4°C). The supernatant was stored at −80°C for cytokines assay. The pellets were resuspended in saline and the total cell and differential cell counts were performed by using the cell counter instrument. The lungs were collected and sliced: one portion to study lung histopathology and second portion for cytokines and hydroxyproline level estimation.

### ELISA test

Quantification of IL-5, IL-6, TNF-alpha, IL-2, and IFN-gamma in BALF and lung homogenate were carried out by using Enzyme-linked immunosorbent assay (ELISA) kit (B.D. Biosciences pharmingen, Bedford, USA), according to the manufacturers protocol. The detection limits for mouse IL-2 and IFN-gamma were 3.2–200 pg/ml whereas 15.6–1000 pg/ml for mouse IL-5, IL-6 and TNF-alpha.

### Histological examination of murine lung tissue

Paraffin-embedded lung tissue was sectioned into 4 μm and dewaxed with xylene. The sections were then stained with hematoxylin-eosinto study cell infiltration, Periodic acid Schiff stain to examine mucus secretion, & Sirius red staining for collagen deposition. Olympus Provis AX70 microscope (Olympus, Lake Success, NY) equipped with a spot RT color digital camera (Diagnostic Instruments, Sterling Heights, MI) were applied to capture the image.

### Quantification of hydroxyproline level

Hydroxyproline is a collagen deposition marker that can be measured in lung homogenate and is indicative of airway remodeling. Deposition of collagen in lungs is indicative of lung fibrosis (Limjunyawong et al., 2014; Srivastava et al., 2016). Samples were treated with alkali for hydrolysis and oxidized with chloramine T to form pyrrole. The addition of Ehrlich's reagent resulted into formation of chromophore that was measured at a bandwidth of 550 nm.

### Statistical analysis

All data were expressed as means ± standard error mean (S.E.M). Total cell counts, differential cell counts, and cytokines in BALF and lung homogenate were analyzed by one-way analysis of variance (ANOVA). Each test was followed by the Dunnets multiple comparison. *P-*values less than 0.05 (*p* < 0.05) were considered statistically significant.

## Results

### Effect of kinase inhibitors on inflammatory cell recruitment in BALF

**Figure 1:**
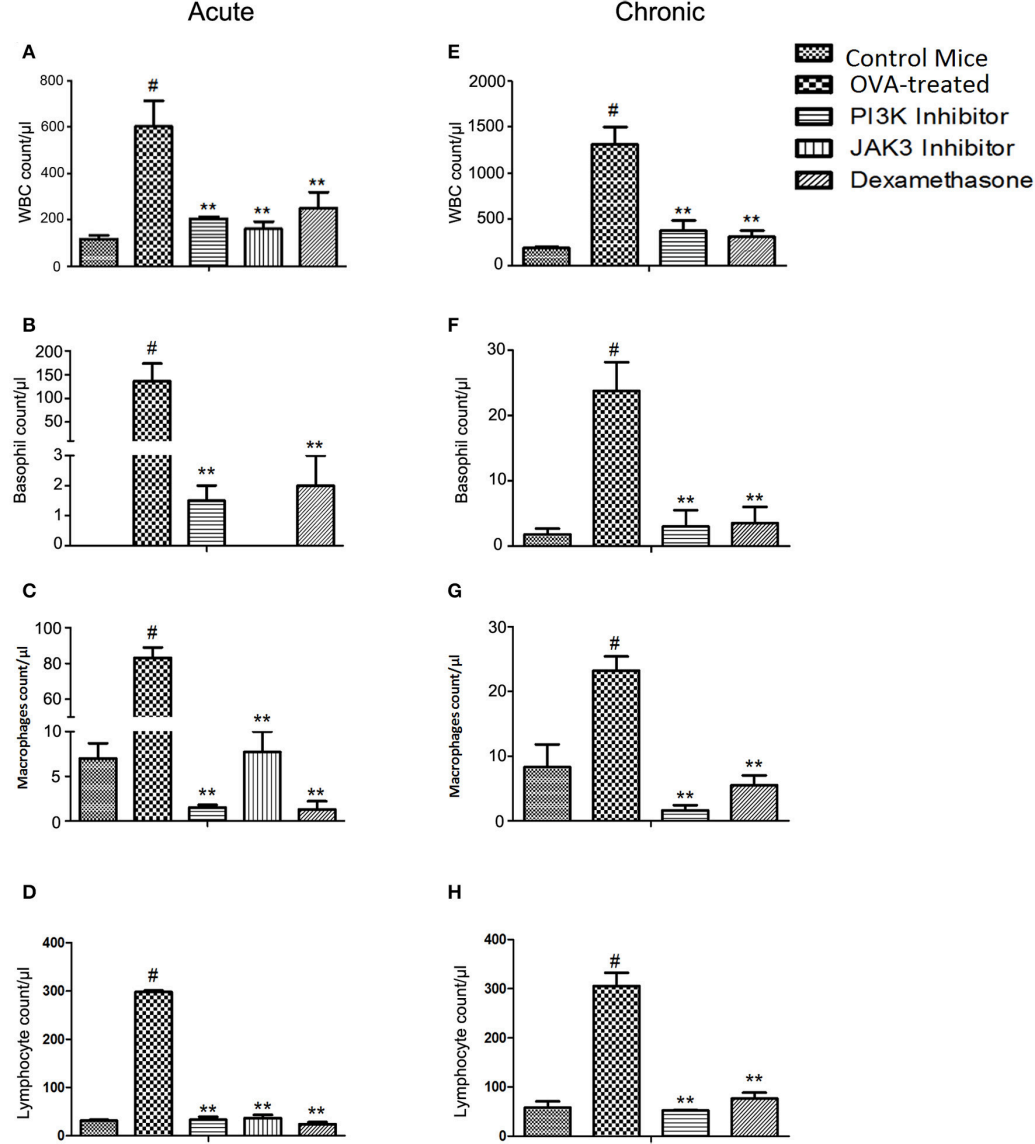
**Effect of treatment on inflammatory cells count in BAL fluid on exposure to kinase inhibitors (30 mg/kg). (A–D)** the cell counts in acute model of asthma after 3 days of treatment. **(E–H)** the cell counts in chronic model of asthma after 10 days of treatment. Data were analyzed by one-way ANOVA followed by Dunnett's multiple comparisons test. Values were expressed as Mean ± S.E.M. (*n* = 6). Statistical significance was assessed as ^**^p < 0.01 vs. OVA-treated group and #*p* < 0.01 vs. control mice group.

OVA-treated mice showed elevation of total inflammatory cells in BALF. On treatment with PI3K and JAK3 inhibitors orally (30 mg/kg), the inflammatory cell counts, such as basophils, neutrophils, macrophages and lymphocytes were reduced to almost normal and *p*-value was statistically significant in acute (Figures 1A–D; Supplementary Figure 1C and Supplementary Table 1) and chronic model of asthma (Figures 1E–H; Supplementary Figure 1D and Supplementary Table 4), except eosinophil level in acute model (Supplementary Figure 1A).

However, in chronic model the level of eosinophil was reduced by 4-fold (Supplementary Figure 1B) with PI3K inhibitor, which indicates oral dosing suppresses eosinophilic inflammation in chronic allergic airway inflammation.

### Effects of kinase inhibitors on cytokines in BALF and lung homogenate in acute and chronic model of asthma

**Figure 2:**
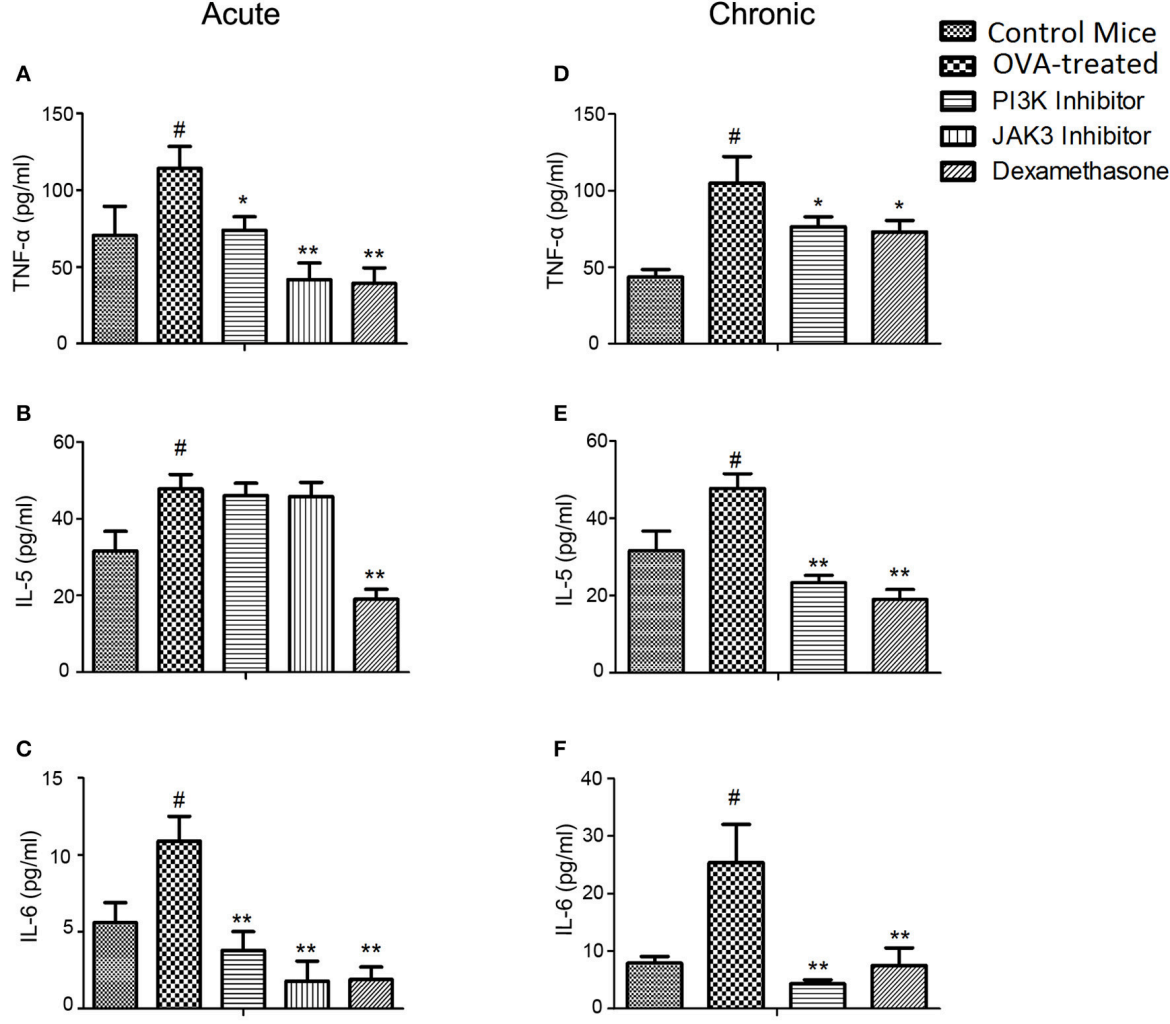
**Effect of treatment on cytokine levels in acute and chronic model of asthma. (A–C)** the level of cytokines (TNF-α, IL-5, and IL-6) were measured in BAL fluid in acute model mice. In chronic model mice, the TNF-α **(D)** from lung homogenate and IL-5 and IL-6 **(E,F)** levels from BAL fluid were determined after 10 days of treatment. Data were analyzed by one-way ANOVA followed by Dunnett's multiple comparisons test. Values were expressed as Mean ± S.E.M. (*n* = 6). Statistical significance was assessed as ^**^p < 0.01, ^*^p < 0.05 vs. OVA-treated group and #*p* < 0.01 vs. control group.

In acute model of asthma, the levels of TNF-α (*p* < 0.05) and IL-6 (*p* < 0.01) in BALF obtained from OVA-treated mice were higher as compared to control mice. Treatments decreased the levels of TNF-α and IL-6 in BALF (Figures 2A–C; Supplementary Table 2) and lung homogenate (Supplementary Figures 2A,C and Supplementary Table 3) compared to OVA-treated mice. Levels of IFN-gamma and IL-2 (Th-1 cell mediated cytokines) were lower in OVA than control mice, but the treatments were ineffective in increasing the level of protective cytokines in both BALF (Supplementary Figures 3A,B and Supplementary Table 2) and lung homogenate (Supplementary Figures 3C,D and Supplementary Table 3). IL-5 (major cytokine responsible for eosinophilic infiltration in asthma) was found to be higher in BALF and lung homogenate of OVA-treated mice as compared to control mice. However, the treatments were ineffective in reducing the IL-5 level in BALF (Figure 2B; Supplementary Table 2) and lung homogenate (Supplementary Figure 2B and Supplementary Table 3).

In asthma model of chronic allergic airway inflammation, the TNF-α (*p* < 0.05) and IL-6 (*p* < 0.01)levels were reduced in BALF and lung homogenate on treatment with kinase inhibitor (Figures 2D–F; Supplementary Figures 2D–F and Supplementary Tables 5, 6). IL-5 level was reduced with a *p*-value of < 0.01 in BALF (Figure 2E; Supplementary Table 5) and lung homogenate (Supplementary Figure 2E and Supplementary Table 6). The protective cytokines (IFN-gamma and IL-2) were not significantly recovered by treatments as observed in BALF (Supplementary Figures 3E,F and Supplementary Table 5) and lung homogenate (Supplementary Figures 3G,H and Supplementary Table 6).

### Effects of kinase inhibitors on hydroxyproline levels in lung homogenate

**Figure 3:**
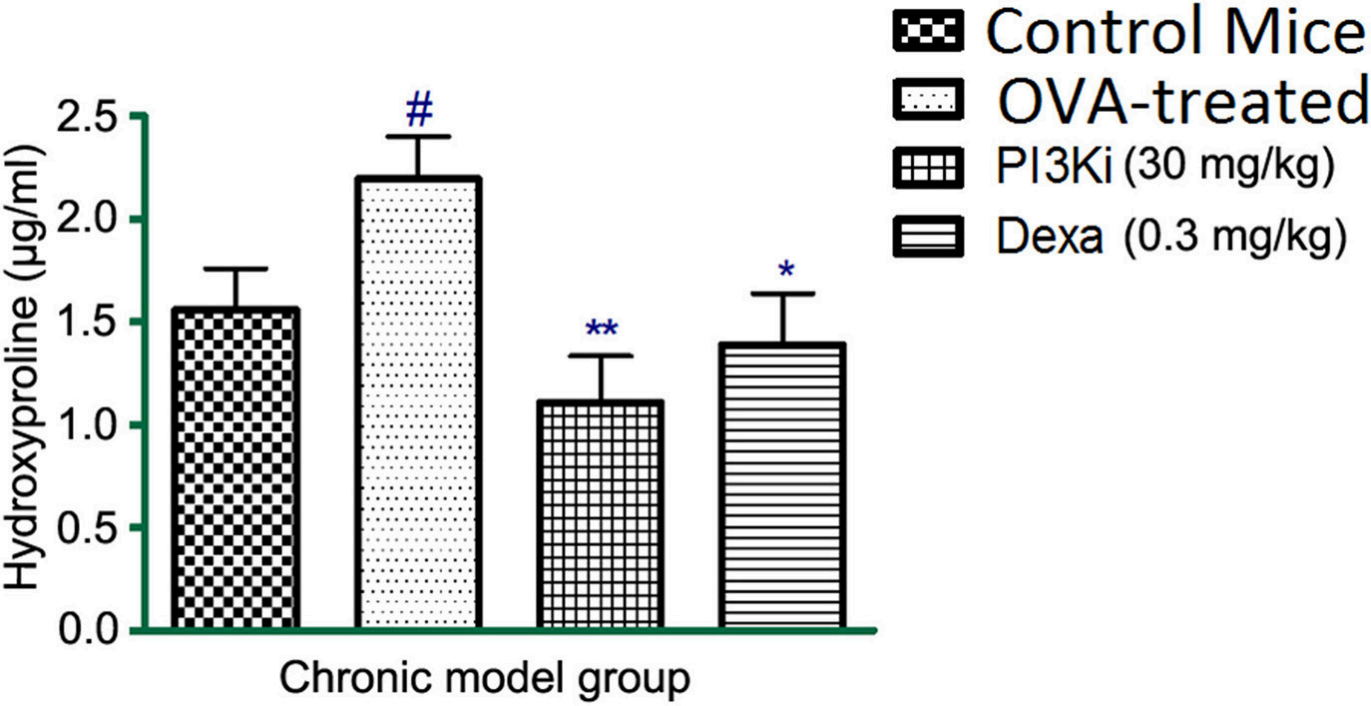
**The effect of different treatments on hydroxyproline levels in lung homogenate after 10 days of treatment**. Data were analyzed by one-way ANOVA followed by Dunnett's multiple comparisons test. Values were expressed as Mean ± S.E.M. (*n* = 6). Statistical significance was assessed as ^**^p < 0.01, ^*^p < 0.05 vs. OVA-treatedgroup and #*p* < 0.01 vs. control mice.

In OVA–treated mice the levels of hydroxyproline was increased as compared to control mice. However, on treatment with PI3K inhibitor, the level of hydroxyproline was reduced in lung homogenate (Figure 3).

### Effects of kinase inhibitors on inflammatory cells and mucus production and collagen deposition

On day 31, the lung tissues were histologically examined. OVA—treated mice were dosed with PI3K inhibitor (30 mg/kg) and dexamethasone (0.3 mg/kg). All the subsequent samples of lung tissues were stored in paraffin to perform the histological analysis.

**Figure 4:**
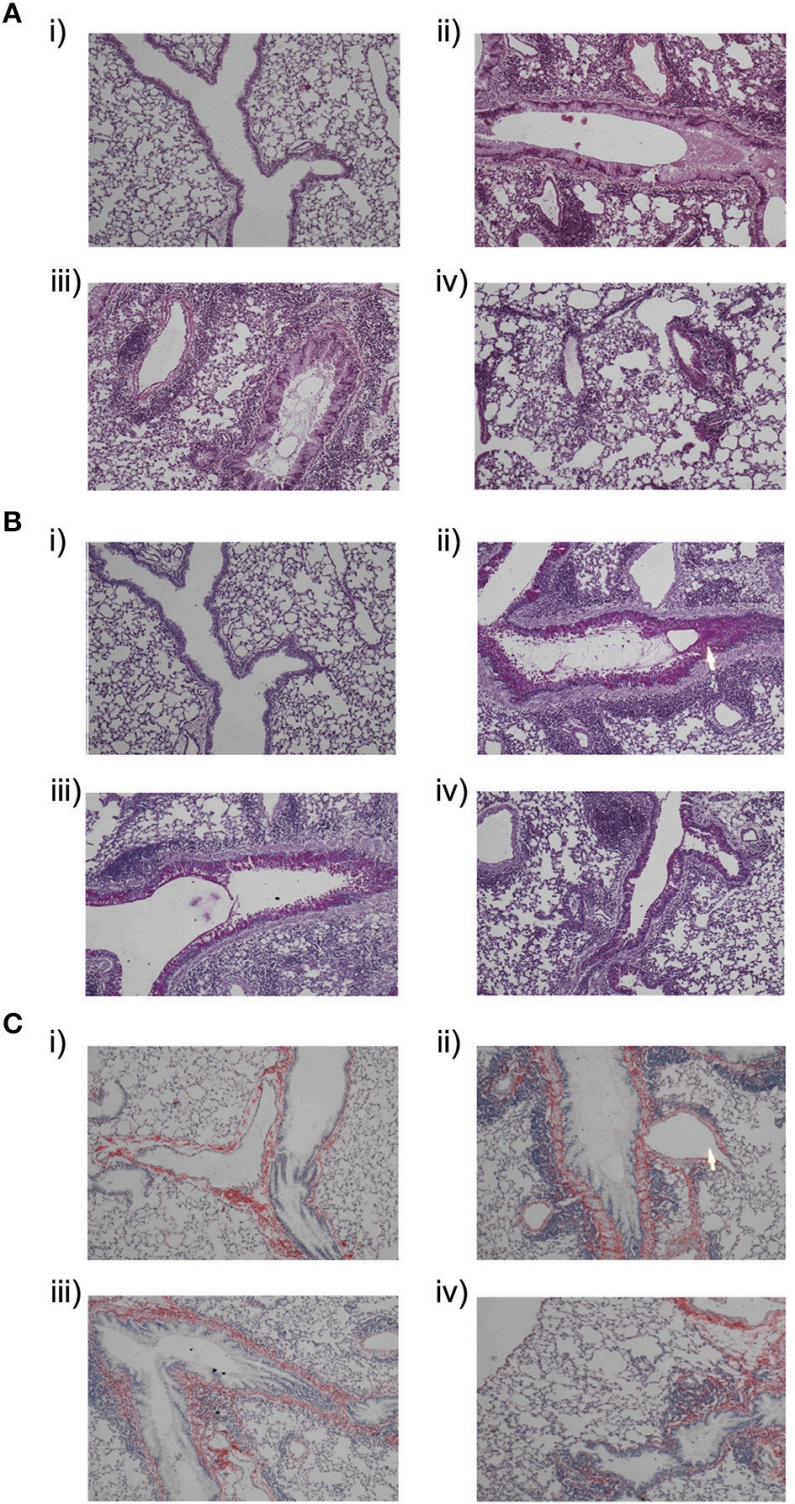
**Histological features of lung tissue sectioned at 5 μm was assessed after staining with (A)** Hematoxylin and eosin staining:**(i)**control mice, **(ii)** OVA-treated, **(iii)** PI3Ki (30 mg/kg, p.o), and **(iv)** Dexamethasone (0.3 mg/kg, p.o), **(B)** Periodic acid–Schiff staining:**(i)**control mice, **(ii)** OVA-treated, **(iii)** PI3Ki (30 mg/kg, p.o), and **(iv)** Dexamethasone (0.3 mg/kg, p.o) and **(C)** Sirius red staining:**(i)** control mice**(ii)** OVA-treated, **(iii)** PI3Ki (30 mg/kg, p.o) and **(iv)** Dexamethasone (0.3 mg/kg, p.o). The arrow indicates the infiltration and mucus secretion. All magnifications were performed at resolution of 40x.

In control mice no inflammatory responses were observed (Figure 4A-i), whereas OVA-treated mice showed extensive infiltration of inflammatory cells around airways and blood vessels (Figure 4A-ii) on hematoxylin and eosin staining lung tissue. The majority of the infiltrated inflammatory cells were eosinophils. On administration of PI3K inhibitor (30 mg/kg; p.o) reduction in inflammatory cells was observed in peribronchial and perivascular region in comparison with OVA-treated mice (Figure 4A-iii). On periodic acid–Schiff staining the presence of goblet cells were seen in OVA sensitized mice. On treatment with kinase inhibitor (30 mg/kg) the hyperplasia of goblet cells were reduced and lower the production of mucus (Figure 4B). Increased airway collagen deposition, a typical feature of airway remodeling in chronic asthma, was assessed peribronchial (Grainge et al., 2011). Sirius red staining indicated the excessive deposition of collagen in the lungs of OVA-treated mice which were reduced in treated mice (Figure 4C).

## Discussion

The present study was proposed to investigate the effect of PI3K and JAK3 inhibitors on airway inflammation characterized by infiltration of inflammatory cells, differential release of cytokines and airway remodeling in acute and chronic model of OVA sensitized mice (Supplementary Figure 4). In this study, we observed significant elevation of inflammatory cells i.e., eosinophil, basophils, neutrophils, macrophages, and lymphocytes in BALF. Our findings are in accordance with earlier findings (Grommes and Soehnlein, 2011; Possa et al., 2013). Thus, the ability to control leukocyte infiltration into lungs is viewed as a key factor in regulating disease severity (Lin et al., 2013).

In acute model of asthma, PI3K and JAK3 inhibitors, and dexamethasone were found effective in inhibiting infiltration of basophils, macrophages, neutrophils, and lymphocytes into bronchoalveolar compartment. In our study we observed increase in basophils infiltration unexpectedly, however these were also attenuated by the treatment. Kinase inhibitors showed dominant inhibitory activity over dexamethasone. JAK3 inhibitor was found highly effective in reducing the neutrophils infiltration as compared to PI3K inhibitor and dexamethasone. However, kinase inhibitors were not effective in inhibiting the eosinophil count, while dexamethasone was found effective. TNF-alpha is a key regulator of pro-inflammatory cytokine and activator of inflammatory cells, which is increased in OVA exposed mice (Zhou et al., 2015). Our results showed kinase inhibitors were effective in inhibiting TNF-alpha in BALF.

Asthma is a Th-2 cell mediated disease, the elevated levels of IL-5 and IL-6 were seen in OVA-treated mice (Deo et al., 2010). In the present study, the level of IL-6 was decreased significantly but not IL-5 in BALF and lung homogenate with kinase inhibitors. Whereas, dexamethasone significantly reduced the IL-5 levels post OVA challenge. Therefore, we observed a clear correlation between cytokine IL-5 level and eosinophil count. IFN-gamma and IL-2 are produced by Th-1 cells which act as a protective mechanism in asthma and their levels were lowered in OVA sensitized mice (Hall and Agrawal, 2014). In this study, the levels of IFN-gamma and IL-2 were restored to normal with JAK3 inhibitor. Level of hydroxyproline was also measured as a biochemical marker of collagen deposition in the lungs (Limjunyawong et al., 2014; Srivastava et al., 2016). An increased level of hydroxyproline in OVA-treated mice was observed, indicating collagen deposition and airway remodeling. However, PI3K inhibitor was found effective in reducing the levels of hydroxyproline in acute model of asthma. From the acute model, we found an overall pronounced effect of JAK3 inhibitor, which is comparable to the first-line therapy drug dexamethasone.

In chronic model of asthma, the PI3K inhibitor was effective in reducing the inflammatory cell count as compared to dexamethasone. Until now, it is reported that the decrease in eosinophil in BALF was correlated with attenuation of asthma symptoms in animal models (Asano et al., 2010). But many clinical studies have also documented a correlation between pulmonary eosinophilia and asthma, and the degree of eosinophil in BALF correlates with disease severity (Ando et al., 2016). Thus, pulmonary eosinophilia has been closely related to asthma symptoms. The suppression of pulmonary eosinophilia by PI3K inhibitor in a chronic model could be explained by the inhibition of both differentiation and migration of eosinophil. IL-5 critically regulates expression of genes that are involved in proliferation, cell survival and maturation of eosinophil (Schwartz et al., 2015). TNF-α may also function as a pro-inflammatory cytokine that causes differentiation and migration of eosinophils (Lin et al., 2013) by recruiting IL-5. Thus, we understand PI3K inhibitor reduces the eosinophils in BALF through suppression of IL-5 cytokines. And our studies suggest that IL-5 is more critical in regulating eosinophils than TNF-alpha.

IFN-gamma and IL-2 levels were significantly increased in BALF with Dexamethasone, but not with PI3K inhibitor in asthma. On treatment with PI3K inhibitor there was decrease in IL-6 level in OVA sensitized mice. In chronic model of asthma, the PI3K inhibitor was effective in controlling airway remodeling as evident by suppressed hydroxyproline level. Histopathology study showed increased infiltration of inflammatory cells, goblet cell hyperplasia, smooth muscle cell hypertrophy and collagen deposition in the OVA-treated mice, which was reduced with PI3K inhibitor. Moreover, the kinase inhibitors were effective in suppressing inflammation and remodeling with no specific side-effects being observed in our study. Recently DP2 receptor antagonists have entered clinical trials for asthma however are faced with challenge to develop once daily treatment with more clinical potency and require further in depth research for use in severe uncontrolled asthma and in pediatric patients with asthma (Santini et al., 2016).

Thus, it was observed that PI3K inhibitor efficiently treats the lung inflammation by inhibiting cell count and cytokine levels. We could not test the JAK3 inhibitor on chronic model in the present study. Recent findings have suggested that selective blocking of bradykinin B_1_ receptor may be an additional therapeutic strategy for the treatment of allergic airway inflammation (El-Kady et al., 2016). In summary, asthmatic models of mice were effectively treated with kinase inhibitor and this could become a closest option for treating allergic asthma.

## Conclusion

In conclusion, kinase inhibitors were able to control the airway inflammation characteristic of bronchial asthma in a murine model of allergic asthma. New kinase inhibitors may yield opportunities for the development of novel therapeutics to treat severe asthma.

## Author contributions

AW, MJ, and VA conceived the study. AW, MS, and JM carried out the animal model construction and the treatment. AW and AC performed the examination of the animal samples. VA reviewed the data and prepared first draft of the manuscript.

### Conflict of interest statement

The authors declare that the research was conducted in the absence of any commercial or financial relationships that could be construed as a potential conflict of interest.

## Abbreviations

PI3K: Phosphoinositide 3 kinase
JAK3: Janus kinase 3
OVA: Ovalbumin
iNOS: Inducible nitric oxide synthase.
