# Supplementary material for: Investigation into the Role of PI3K and JAK3 Kinase Inhibitors in Murine Models of Asthma
Source: Front Pharmacol. 2017 Feb 28;8:82. doi: 10.3389/fphar.2017.00082 (PMC5328984; doi:10.3389/fphar.2017.00082)
Supplement: Supplementary file 1 [file Table1.PDF]

Supplementary table 1: Cell counts in acute asthma

Group	Treatment	Dose (p.o)	WBC/ µl	Eosinophil	Basophil	Neutrophil	Macrophage	Lymphocyte
1.	Normal control	NA	116.6 ±14.4	19.2 ± 1.8	Not detected	57 ± 9.9	7 ± 1.7	31 ± 2.2
2.	OVA control	NA	602.4 ± 111.8	26 ± 1.4	136 ± 37.1	137.2 ± 11.3	83.2 ± 5.8	298 ± 3.2
3.	PI3K inhibitor	30 mg/kg	204.2 ± 9.3	23.5 ± 1.5	1.5 ± 0.5	113.5 ± 14.1	1.5 ± 0.3	33 ± 6.1
4.	JAK3 inhibitor	30 mg/kg	158.7 ± 33.0	24 ± 1.5	Not detected	73.25 ± 14.5	7.7 ± 2.3	36.7 ± 6.6
5.	Dexamethas one	0.3 mg/kg	249 ± 69.8	13 ± 2	2 ± 1	107 ± 13.3	1.3 ± 0.9	24 ± 4
