# Supplementary material for: Investigation into the Role of PI3K and JAK3 Kinase Inhibitors in Murine Models of Asthma
Source: Front Pharmacol. 2017 Feb 28;8:82. doi: 10.3389/fphar.2017.00082 (PMC5328984; doi:10.3389/fphar.2017.00082)
Supplement: Supplementary file 2 [file Table2.PDF]

Supplementary table 2: Cytokines of acute asthma in BALF

Group	Treatment	Dose (p.o)	TNF-α (pg/ml)	IL-6	IL-5	IL-2	IFN-gamma
1.	Normal control	NA	70.5 ± 19.0	5.6 ± 1.3	31.6 ± 5.1	71.6 ± 8.3	121.1 ± 21.9
2.	OVA control	NA	114.2 ± 14.3	10.9 ± 1.6	47.8 ± 3.8	63.4 ± 7.1	80.7 ± 7.7
3.	PI3K inhibitor	30 mg/kg	73.9 ± 8.8	3.8 ± 1.2	46.1 ± 3.2	52.3 ± 4.8	58.3 ± 7.9
4.	JAK3 inhibitor	30 mg/kg	41.8 ± 10.8	1.8 ± 1.3	45.8 ± 3.7	40.2 ± 8.5	41.6 ± 7.8
5.	Dexamethasone	0.3 mg/kg	39.3 ± 10.1	1.9 ± 0.8	19 ± 2.6	55.5 ± 5.3	106.9 ± 18.4
