# Supplementary material for: Investigation into the Role of PI3K and JAK3 Kinase Inhibitors in Murine Models of Asthma
Source: Front Pharmacol. 2017 Feb 28;8:82. doi: 10.3389/fphar.2017.00082 (PMC5328984; doi:10.3389/fphar.2017.00082)
Supplement: Supplementary file 3 [file Table3.PDF]

Supplementary table 3: Cytokines of acute asthma in Lung Homogenate

**Group**	**Treatment**	**Dose (p.o)**	**TNF-α (pg/ml)**	**IL-6**	**IL-5**	**IL-2**	**IFN-gamma**
1.	Normal control	NA	36.01 ± 5.1	7.9 ± 1.4	38.1 ± 0.5	167.8 ± 33.5	759.5 ± 108.5
2.	OVA control	NA	78.7 ± 3.7	16.6 ± 1.5	64.7 ± 10.8	131.7 ± 3.6	57.1 ± 15.1
3.	PI3K inhibitor	30 mg/kg	91.4 ± 5.8	5 ± 1.5	80.7 ± 7.3	136.5 ± 6.8	69.1 ± 20.1
4.	JAK3 inhibitor	30 mg/kg	86.6 ± 6.9	5.6 ± 0.99	62.8 ± 14.6	170.1 ± 8.7	218.5 ± 92.3
5.	Dexamethasone	0.3 mg/kg	55.5 ± 15.3	4.33 ± 0.33	59.1 ± 1.1	104.4 ± 15.1	89.7 ± 41.1
