# Supplementary material for: Investigation into the Role of PI3K and JAK3 Kinase Inhibitors in Murine Models of Asthma
Source: Front Pharmacol. 2017 Feb 28;8:82. doi: 10.3389/fphar.2017.00082 (PMC5328984; doi:10.3389/fphar.2017.00082)
Supplement: Supplementary file 4 [file Table4.PDF]

Supplementary table 4: Cell counts of chronic asthma

Group	Treatment	Dose (p.o)	WBC/µl	Eosinophil	Basophil	Neutrophil	Macrophage	Lymphocyte
1.	Normal control	NA	193.8 ± 13.5	27.5 ± 3.1	1.8 ± 0.9	90 ± 13.3	8.3 ± 3.5	58.3 ± 12.4
2.	OVA control	NA	1313.5 ± 193.3	82.4 ± 18.8	23.8 ± 4.4	1367.5 ± 136.7	23.2 ± 2.2	350.4 ± 27
3.	PI3K inhibitor	30 mg/kg	389.7 ± 107.5	19.5 ± 4.3	3 ± 2.51	207.3 ± 59.2	1.6 ± 0.8	52.3 ± 1.2
4.	Dexamethasone	0.3 mg/kg	319.3 ± 66.4	13 ± 1.5	3.5 ± 2.5	175.3 ± 17.8	5.5 ± 1.5	76.6 ± 12
