# Supplementary material for: Investigation into the Role of PI3K and JAK3 Kinase Inhibitors in Murine Models of Asthma
Source: Front Pharmacol. 2017 Feb 28;8:82. doi: 10.3389/fphar.2017.00082 (PMC5328984; doi:10.3389/fphar.2017.00082)
Supplement: Supplementary file 5 [file Table5.PDF]

Supplementary table 5: Cytokines of chronic asthma in BALF

**Group**	**Treatment**	**Dose (p.o)**	**TNF-α (pg/ml)**	**IL-6**	**IL-5**	**IL-2**	**IFN-gamma**
1.	Normal control	NA	81.9 ± 12.7	7.9 ± 11.7	31.6 ± 5.1	57.2 ± 8.9	119.7 ± 12.1
2.	OVA control	NA	51.4 ± 8.2	25.4 ± 6.6	47.8 ± 3.8	23.3 ± 2.7	46.9 ± 11.8
3.	PI3K inhibitor	30 mg/kg	36.1 ± 2.9	4.3 ± 0.7	23.4 ± 1.9	31.2 ± 3.6	31.5 ± 4.1
4.	Dexamethasone	0.3mg/kg	82.8 ± 21.1	7.5 ± 3.08	19 ± 2.6	50 ± 9.2	90.8 ± 13.6
