# Supplementary material for: Investigation into the Role of PI3K and JAK3 Kinase Inhibitors in Murine Models of Asthma
Source: Front Pharmacol. 2017 Feb 28;8:82. doi: 10.3389/fphar.2017.00082 (PMC5328984; doi:10.3389/fphar.2017.00082)
Supplement: Supplementary file 6 [file Table6.PDF]

Supplementary table 6: Cytokines of chronic asthma in Lung Homogenate

**Group**	**Treatment**	**Dose (p.o)**	**TNF-α (pg/ml)**	**IL-6**	**IL-5**	**IL-2**	**IFN-gamma**
1.	Normal control	NA	43.5 ± 5.1	7.9 ± 1.4	104.3 ± 26.5	130.9 ± 22.7	625.3 ± 97.9
2.	OVA control	NA	104.9 ± 17.4	16.6 ± 1.5	165.06 ± 17.7	129.7 ± 9.2	59.5 ± 16.2
3.	PI3K inhibitor	30 mg/kg	76.4 ± 6.5	5 ± 1.6	78.8 ± 10.3	121.1 ± 3.1	47.1 ± 8.9
4.	Dexamethasone	0.3 mg/kg	73.1 ± 7.4	4.3 ± 0.3	120.5 ± 3.9	112.6 ± 9.8	30.8 ± 8.2
