# Supplementary material for: Investigation into the Role of PI3K and JAK3 Kinase Inhibitors in Murine Models of Asthma
Source: Front Pharmacol. 2017 Feb 28;8:82. doi: 10.3389/fphar.2017.00082 (PMC5328984; doi:10.3389/fphar.2017.00082)
Supplement: Supplementary file 7 [file Image1.PDF]

Supplementary Figure 1

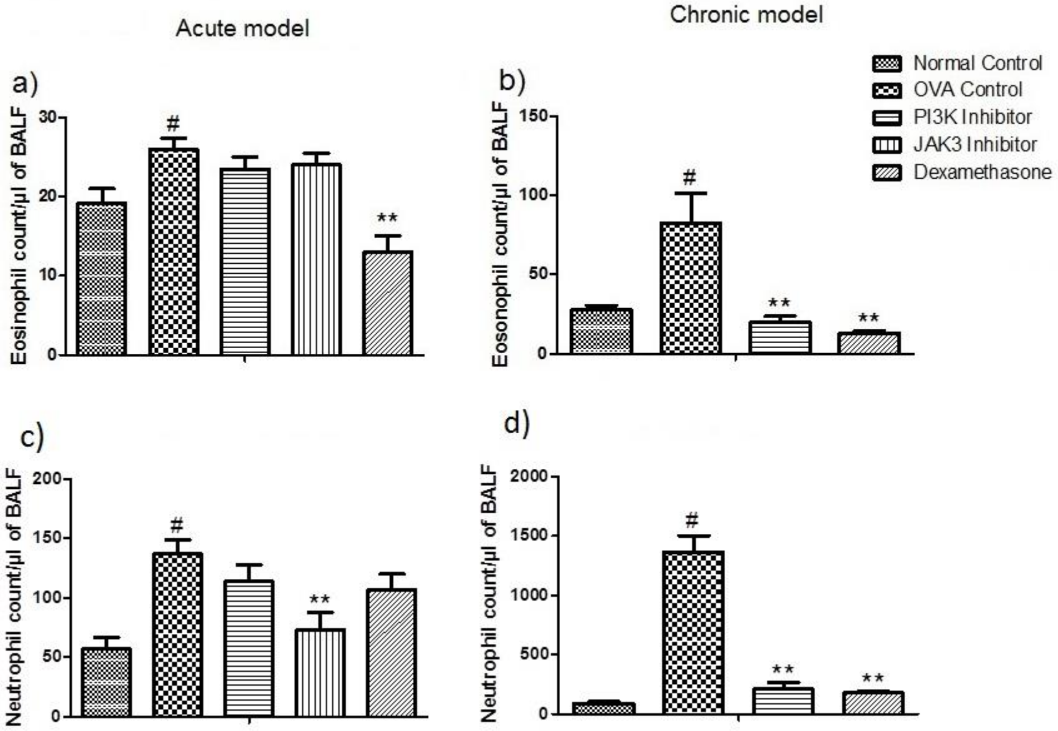

Supplementary figure 1: Eosinophil and Neutrophil cells count in BALF on expose to kinase inhibitors (30mg/kg). **(1a and 1c)** the level of cell counts in acute asthma after 3 days of treatment. **(1b and 1d)** the level of cell counts in chronic asthma after 10 days of treatment. Data were analysed by one-way ANOVA followed by Dunnett's multiple comparisons test. Values were expressed as Mean ±S.E.M. (n=6). Statistical significance was assessed as **: $p < 0.01$ Vs OVA control group and #: $p < 0.01$ vs Normal control group.
