# Supplementary material for: Investigation into the Role of PI3K and JAK3 Kinase Inhibitors in Murine Models of Asthma
Source: Front Pharmacol. 2017 Feb 28;8:82. doi: 10.3389/fphar.2017.00082 (PMC5328984; doi:10.3389/fphar.2017.00082)
Supplement: Supplementary file 8 [file Image2.PDF]

Supplementary figure 2

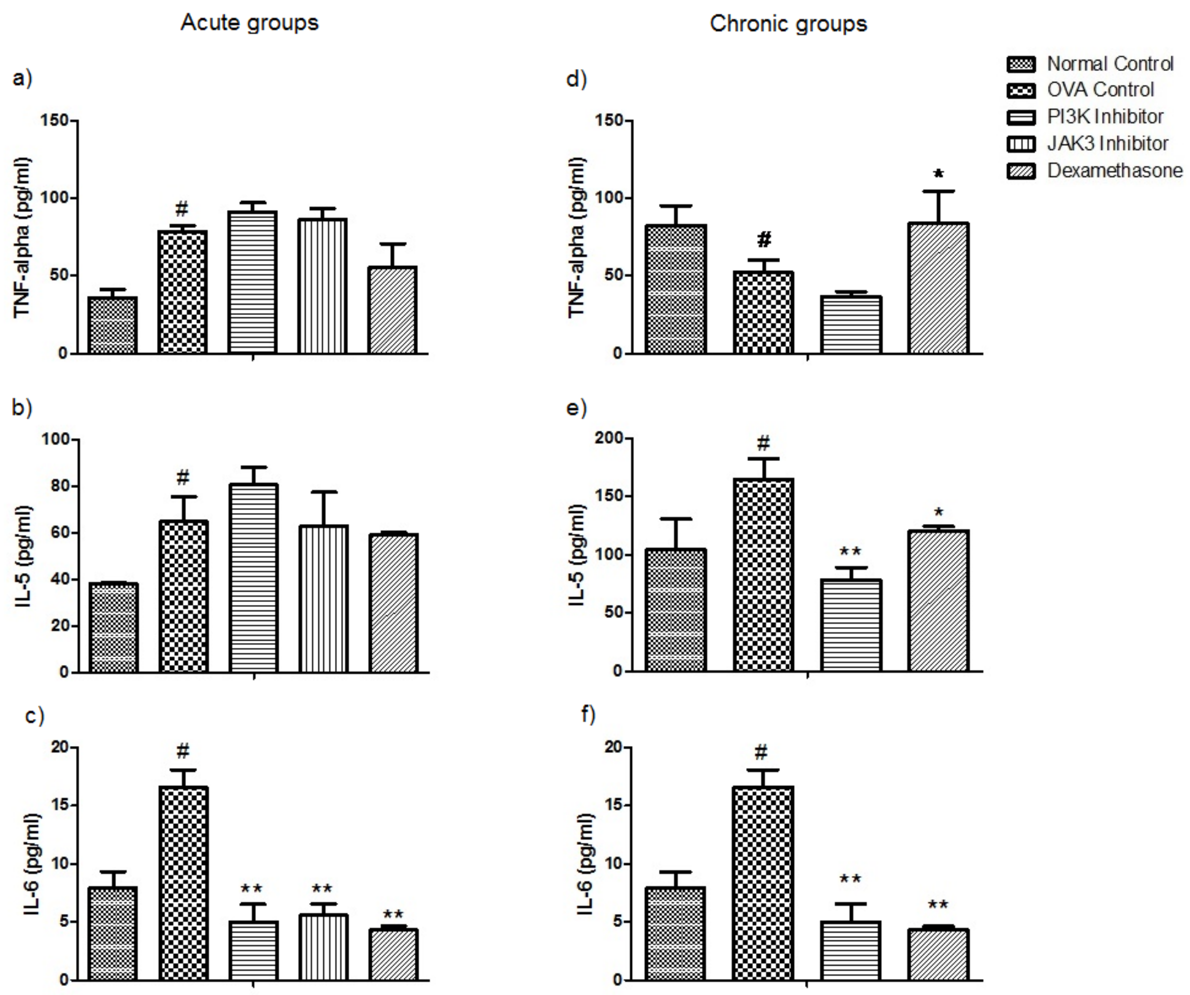

Supplementary figure 2: Effect of treatment on inflammatory cytokines level in acute and chronic model of asthma. **(a-c)** the level of cytokines (TNF-α, IL-5 and IL-6) were measured in lung homogenate in acute mice after 3 days of initial treatment. In chronic mice, the TNF-α **(d)** from BALF, IL-5 and IL-6 **(e and f)** levels from lung homogenate were determined after 10 days of initial treatment. Data were analyzed by one-way ANOVA followed by Dunnett's multiple comparisons test. Values were expressed as Mean ± S.E.M. (n=6). Statistical significance was assessed as **: $p < 0.01$, *: $p < 0.05$ Vs OVA control group and #: $p < 0.01$ vs Normal control group.
