# Supplementary material for: Investigation into the Role of PI3K and JAK3 Kinase Inhibitors in Murine Models of Asthma
Source: Front Pharmacol. 2017 Feb 28;8:82. doi: 10.3389/fphar.2017.00082 (PMC5328984; doi:10.3389/fphar.2017.00082)
Supplement: Supplementary file 9 [file Image3.PDF]

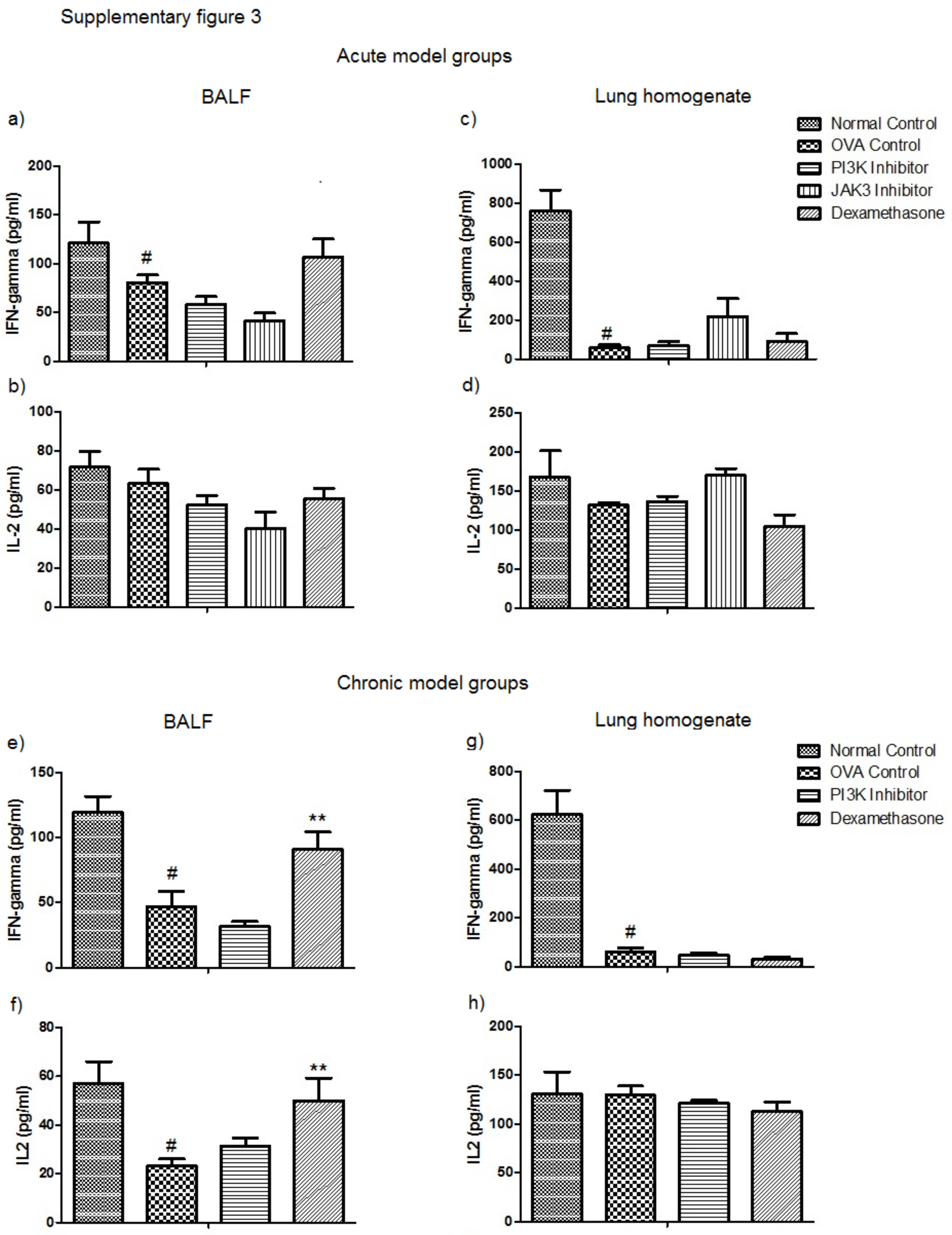

Supplementary figure 3: Effect of treatment on protective cytokines in OVA sensitized mice. **(a-d)** the level of cytokines (IFN-gamma and IL-2) were measured in BALF and lung homogenate in acute mice after 3 days of initial treatment. **(e-h)** In the level of cytokines (IFN-gamma and IL-2) were measured in BALF and lung homogenate in chronic mice after

10 days of initial treatment. Data were analyzed by one-way ANOVA followed by Dunnett's multiple comparisons test. Values were expressed as Mean ± S.E.M. (n=6). Statistical significance was assessed as **: $p < 0.01$, *: $p < 0.05$ Vs OVA control group and #: $p < 0.01$ vs Normal control group.
