# Supplementary material for: Investigation into the Role of PI3K and JAK3 Kinase Inhibitors in Murine Models of Asthma
Source: Front Pharmacol. 2017 Feb 28;8:82. doi: 10.3389/fphar.2017.00082 (PMC5328984; doi:10.3389/fphar.2017.00082)
Supplement: Supplementary file 10 [file Image4.PDF]

Supplementary figure 4

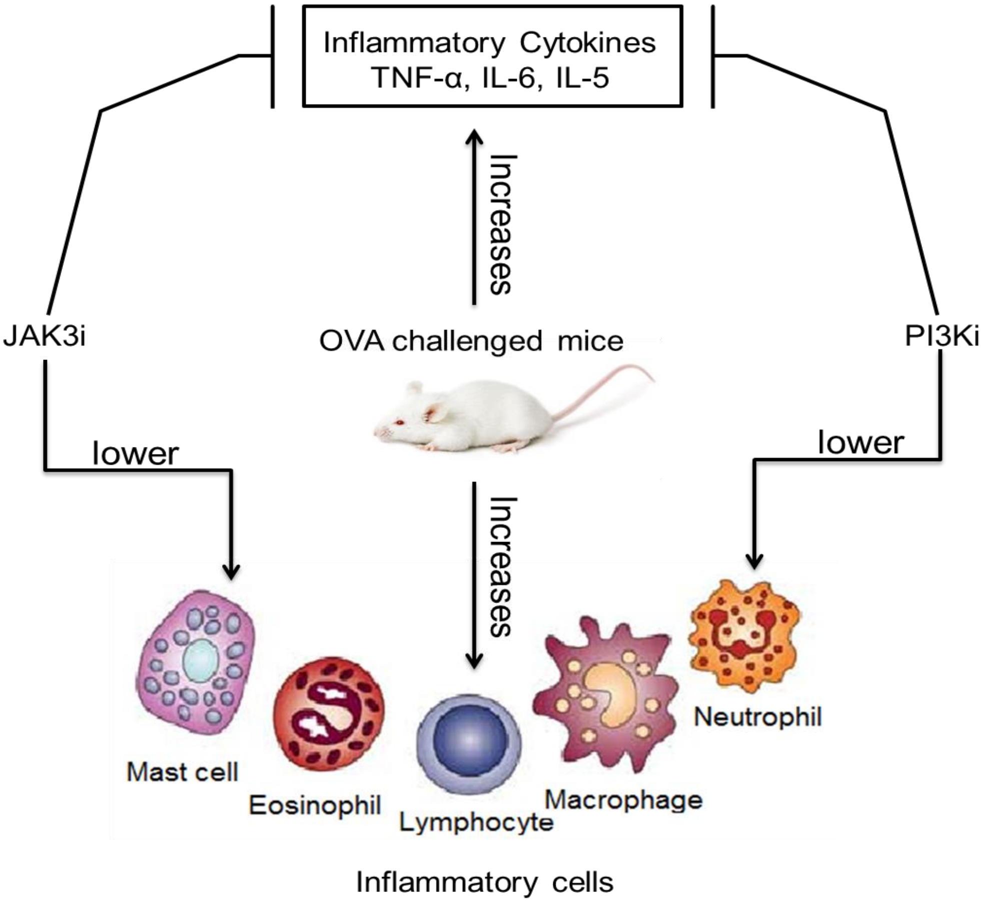

Supplementary figure 4: Overview of PI3K and JAK3 inhibitor activity in ovalbumin challenged mice.
